# Influence of Using Various Levels of Protein Concentrate in Rations of Ayrshire Dairy Cows on Rumen Microbiome, Reproductive Traits and Economic Efficiency

**DOI:** 10.3390/vetsci9100534

**Published:** 2022-09-28

**Authors:** Nikolai P. Buryakov, Dmitrii E. Aleshin, Maria A. Buryakova, Anastasya S. Zaikina, Georgy Y. Laptev, Larisa A. Ilina, Aleksandr S. Petrov, Nikolay M. Kostomakhin, Ahmed I. El Sheikh, Ferial M. Sahwan, Mohamed M. Fathala

**Affiliations:** 1 Department of Feeding Animals, Institute of Animal Science and Biology, Russian State Agrarian University—Moscow Timiryazev Agricultural Academy, 127434 Moscow, Russia; 2 Molecular Genetic Laboratory, BIOTROF+ Ltd., 196650 Saint-Petersburg, Russia; 3 Department of Large Animal Husbandry, Faculty of Bioengineering and Biotechnology, Saint-Petersburg State Agrarian University, Pushkin, 196601 Saint-Petersburg, Russia; 4 Department of Veterinary Public Health, College of Veterinary Medicine, King Faisal University, Al-Hofuf 31982, Saudi Arabia; 5 Animal Husbandry and Wealth Development Department, Faculty of Veterinary Medicine, Alexandria University, Alexandria 5410012, Egypt

**Keywords:** rumen microbiome, ration, protein, reproduction ability, Ayrshire breed, economic efficiency

## Abstract

**Simple Summary:**

Numerous studies in animal nutrition science have demonstrated a close connection between the chemical composition and nutritional value of cow diets, the number of rumen bacterial communities and animal productive and reproductive performance. Thus, the microorganisms of cow rumen play a primary role in the processes of digestion; meanwhile, it is necessary to highlight that their functional significance is much wider. The aim of this study was to investigate the outcomes of the inclusion of different levels of protein concentrate in rations of Ayrshire dairy cows in relation to the rumen microbiome, reproductive traits and economic value. Our manuscript presents molecular biological studies to establish the quantitative and specific composition of the microorganisms of the rumen. T-RFLP analysis was performed to determine the percentage of the components of the bacterial community. The results showed that the supplementation of “Agro-Matic” caused no deviations from the normal standards of cellulolytic, amylolytic, transit and pathogenic bacteria, no impact on reproductive traits and significantly improved the profitability of the milk production process. These findings provide a scientific addition for a greater understanding of the rumen microbiome of dairy cows when supplemented by different levels of protein concentrate in their rations.

**Abstract:**

Animal feeding research has revealed a close relationship between the chemical composition and nutritional value of cow rations, the number of rumen bacterial communities and animal productivity. Our present research aimed to investigate the outcome of inclusion of different levels of protein concentrate in rations of Ayrshire dairy cows in relation to the rumen microbiome, reproductive traits and economic value. Forty-five Ayrshire cows were divided into three groups (15 in each). The first control group 0 AM was fed the basal ration, while the second 1 AM and third 2 AM groups were fed the basic ration with the sunflower cake replaced by different levels of protein concentrate Agro-Matic (1 and 1.5 kg/head/day, respectively). Ruminal fluid samples, reproductive parameters and economic value were studied. During the early lactation period, 120 days in milk (DIM), the number of pathogenic microorganisms decreased in both the 1 AM and 2 AM groups when compared with the control group 0 AM; moreover, a significant decrease in Peptococcus bacteria was recorded in the 1 AM group, while Fusobacterium decreased in the 2 AM group. At the end of lactation, the total number of cellulolytic bacteria increased with the use of protein concentrate in animals of the 1 AM group when compared with the control group. Regarding undesirable bacteria, the 2 AM group recorded the highest value for Lactobacilli and Actinobacteria when compared with the 0 AM group (0.18 and 8.90 vs. 0.04 and 4.24), and the differences were significant (*p* < 0.05). The insemination index and the duration of the days open period decreased in the 2 AM group, while the differences were *p* > 0.05. The profitability of milk production increased by 2.76% and 6.28% in both supplemented groups, and the differences compared to the 0 AM group were significant. We conclude that the supplementation of Agro-Matic caused no deviations from the normal standards of cellulolytic, amylolytic, transit and pathogenic bacteria, no impact on reproductive functions and significantly improved the profitability of the milk production process of Ayrshire dairy cows.

## 1. Introduction

Studies on animal feeding have shown a close association between the chemical composition and nutritional value of the ration of cows, the number of bacterial communities of the rumen and the productivity of animals [1,2,3,4]. Thus, the microorganisms of cow rumen play a primary role in the processes of digestion; meanwhile, it is necessary to emphasize that their functional significance is much broader. The symbiotic microflora of the rumen perform a detoxification function, participate in the digestibility of nutrients, prevent the invasion of parasites and pathogens and stimulate the development of immunity [4,5,6]. Therefore, the maintenance and functioning of the microbiota are also important factors in the prevention of gastrointestinal diseases, such as lactate acidosis, alkalosis, ketoses, laminates, mastitis and limb diseases, which significantly increase the productivity of dairy cattle [2,3,7,8,9,10,11,12,13,14].

Wang et al. (2018) [11] used the 16S rRNA gene sequencing method for studying the goat rumen microbial community. They found that healthy animals (goats) had a similar taxonomic profile, while significant differences were found in goats suffering from diarrhea manifestations. In particular, animals with diarrhea showed a decrease in species richness and diversity. In another study, Zhong et al. (2014) [12] demonstrated the association between the composition of the rumen microbiota and the number of somatic cells in milk. They found that cows with clinical manifestations of mastitis and a high number of somatic cells in their milk had an increased rumen bacterial diversity and an increased proportion of bacteria phylum SR1, Actinobacteria, Clostridiales and Butyrivibrio.

Dairy cattle feed includes the participation of anaerobic microorganisms with undetermined enzymatic activity that live in the rumen and other parts of the gastrointestinal tract of the animal [14,15,16]. Thus, the study of rumen content and regulation of the microbial population to increase the milk productivity of cows becomes practical in dairy and beef cattle breeding [15,17,18]. Throughout the process of the growth and development of ruminants and certain anaerobic microorganisms, which develop when feed and water are consumed, symbiotic associations are developed in the rumen. The developed symbiotic relationship between the host and the microbiota results in mutually beneficial cooperation [6,19,20].

Sunflower cake and meal are obtained by pressing and extracting oil from sunflower seeds [21]. Nevertheless, it should be noted that they experience a high degree of protein breakdown in the rumen. Thus, the degree of the degradable protein is 97%, and only 3% is non-degradable fractions that enter the small intestine where they are broken down to amino acids and used by the animal body for milk synthesis [21,22,23,24]. The content of crude protein in sunflower and soybeans meal varies significantly (26–50%), and crude fiber comprises 15–33% [22,23,24]. As a result of the flow digestibility of the sunflower meal and due to the high fiber content, it is fed to cows at no more than 3.6 kg/head/day. Therefore, the production practice of the developed countries of the world has shown that lupin is now the most promising crop of legumes [25,26]. It is a high-protein crop which is slightly inferior to soy in terms of the content of metabolic energy, crude fat and the biological value of the protein [27].

The protein content in lupin averages 50–60%, while a large proportion of the protein is represented by globulins, albumins (26–40%) and glutamins (12%). The biological value of lupin protein is 78% [23,24,26,27], and, in terms of the total content of essential amino acids, lupin (289.0 g/kg CP) is slightly inferior to soy (332.7 g/kg CP) and surpasses feed beans (134.9 g/kg CP), which are at the same level. Peas occupy last place, and the content of essential amino acids is 99.1 g/kg in natural grain [23,27]. At the same time, the content of methionine, cystine, leucine and isoleucine in white lupin grain is higher than in soybeans [28,29,30], but it is deficient in tryptophan [5,23,24,27]. The protein of white lupin grain consists of 36.7% essential amino acids, where 5.9–7.1% is allocated to the content of leucine, 4.1–7.5% to lysine and 3.1–4.3% to valine [25,29].

The processing of such resources with a protein content of 20% or more, using modern research, has increased the production of food and feed protein to 5 million tons annually, which makes up for its deficiency [30,31,32]. However, the high price of feed and the undulation in the production of animal feed promote the search for alternative protein sources that can be used in the diets of ruminant animals [32,33,34,35]. Consequently, this research was carried out to study the bacterial community of the rumen of lactating cows; evaluate some indicators of the reproductive ability of cows; calculate the economic efficiency of using protein concentrate Agro-Matic in the feed of Ayrshire lactating cows; and give recommendations on the use of the protein concentrate Agro-Matic in the diet of highly productive cows during production.

## 2. Materials and Methods

### 2.1. Animals and Feed Strategy

The current study was carried out from March 2018 to 2021 on a farm belonging to Agricultural Production Cooperative ‘Plemzavod Maysky’ in the Vologda region, Russia. The research was carried out in accordance with the methodology approved by the bioethics committee of the Russian State Agrarian University Russian State Agrarian University - Moscow Timiryazev Agricultural Academy (protocol no. 2021-4, 12 October 2021).

Forty-five Ayrshire dairy cows in dry period (three weeks before calving) were selected and divided into 3 groups (15 in each), taking into account the origin, age, live weight and physiological state, with a milk productivity of more than 8000 kg of milk per lactation. The animals were clinically healthy and were housed under the same conditions of housing and management throughout the experiment (3 weeks before calving until the end of lactation period). Cows were milked three times per day (3X).

The first group of cows, which served as control group 0 AM, received the normal basic ration after balancing the nutrient contents as per the requirements of high-yielding cows (All-Russia Institute of Animal Husbandry, 2016) [36] (Table 1). On the other hand, cows of the 2nd and 3rd group received normal basic ration with the sunflower cake replaced with 300 gm and 500 gm of protein concentrate Agro-Matic, respectively, until calving. After calving, these cows of the 2nd and 3rd group received normal basic ration with the sunflower cake replaced with 1 and 1.5 kg of protein concentrate Agro-Matic, respectively, until the end of the lactation period.

### 2.2. Feed Composition

The protein concentrate Agro-Matic used consisted of white lupin grain, poultry meat and bone meal (without feathers) (Table 2, Table 3 and Table 4). Amino acids in the Agro-Matic protein concentrate were determined according to state standard 32195-2013 (ISO 13903: 2005) [37], while minerals, except cobalt, were measured by atomic absorption spectrometry in accordance with state standard 32343–2013 [38]. Cobalt was determined by photometric method in accordance with state standard 26573.2-2014 [39] in the testing center of the All-Russian State Center for Quality and Standardization of Veterinary Drags and Feed (Moscow). The rations formulation was carried out using the Feed Optima program to fulfill the cows’ requirements (energy, protein, vitamins and minerals) during the dry period and lactation period.

### 2.3. Bacterial Community

In order to study the bacterial community, samples of ruminal fluid were extracted 3 h after feeding using a transesophageal tube (rumen probe) according to the method of Geishauser et al. (2012) [40], Laptev et al. (2016) [7] and Larsen et al. (2020) [22].

Molecular biological studies to establish the quantitative and specific composition of the microorganisms of the rumen were carried out in the laboratory of molecular genetics at BIOTROF+ LLC. T-RFLP analysis was performed to determine the percentage of components of the bacterial community. The T-RFLP analysis method is one of the most popular metagenomic methods of the first generation, since it is a fast and fairly easily reproducible way to assess changes in natural and artificial microbial communities [41].

This method is based on the analysis of the variability of the conservative genome regions of the 16S DNA genes of the bacteria of the rumen content of experimental cows since it has been established that the certain region of the gene that enters (encodes) 16S rRNAs contains highly conserved sequences and hypervariable regions. Due to this, we can use primers for sequencing complementary to conservative sites to obtain sequences of hypervariable sites that can classify (intensify) a microorganism to a specific species [42].

Microflora analysis by the T-RFLP method includes the following stages:

1. Allocation of the total (total) DNA of microorganisms;
2. PCR amplification of fragments of bacterial genes (16S rDNA) with fluorescently labeled primers (from the 5′ end);
3. Enzymatic treatment of the amplification using restriction endonucleases (endonucleases that recognize sequences of nucleotides are usually used);
4. Separation of DNA fragments obtained as a result of restriction in a polyacrylamide gel in a sequencer together with a fluorescently labeled DNA marker of known size.

Total DNA from the samples was isolated using the “Genomic DNA Purification Kit” (Fermentas, Inc., Vilnius, Lithuania) according to the manufacturer’s recommendations. PCR was performed on a Verity DNA amplifier (Life Technologies Corp., Inc., Carlsbad, USA) with eubacterial primers (Table 5), which allowed the amplification of a fragment of the 16S rRNA gene with positions from 63 to 1087 (numbering is indicated for the 16S rRNA gene *Esherichia coli*) in the mode: 95 °C—3 min (1 cycle); 95 °C—30 s, 55 °C—40 s, 72 °C—60 s (35 cycles), 72 °C—5 min.

The calculation of the size of the peaks and their area was carried out in the Fragment Analysis program (Life Sciences, Indianapolis, USA) and was accepted in the study on the basis of which subtypes (phylotypes) were isolated with an error of 1 nucleotide, and their relative content in the microbial community was determined. The belonging of bacteria to a certain taxonomic group was determined using a database (http://mica.ibest.uidaho.edu/trflp.php, accessed on 22 January 2022).

### 2.4. Reproductive Ability

The following parameters were estimated to assess the reproductive ability of dairy cows.

Calving interval was estimated as the time interval between two successive calvings, and days open period was estimated as the time interval between the date of calving and date of successful conception and service per conception (insemination index per conception).

### 2.5. Economic Efficiency

The economic efficiency analysis was carried out at the end of the early lactation period (120 DIM). At the same time, the costs of feed and feed additives were taken into account in the quotes of current prices for the duration of the experiment (2021). The data and parameters included in calculation of economic efficiency were subjected to the same statistical analysis applied to all the studied parameters of the experiment. The economic efficiency was estimated according to Kutikov and Ambrosov (1966) [43] and Antonova et al. (2011) [44] using the following formulas:

Feed costs for the period of experiment = the cost of a daily ration × 120 (DIM);

Cash obtained (total return) from the sale of milk = gross milk yield, kg (120 DIM) × the selling price of 1 kg of milk;

Profit from the sale of milk (net return) = cash obtained from the sale of milk (total return) − total milk production costs;

The profitability % = profit from the sale of milk (net return) / total milk production costs × 100.

### 2.6. Statistical Analysis

Data were statistically analyzed by a one-way analysis of variance test using the SPSS program (2017) [45]. Differences between means were tested according to Duncan’s multiple-range test (Duncan, 1955) [46] at *p* < 0.05 according to the following model:

Xijk = μ + Ai + eijk

where:

Xijk = an individual observation;
μ = overall mean;
Ai = effect of ith treatment (0 AM, 1 AM and 2 AM);
eijk = random error.

## 3. Results

### 3.1. Bacterial Community of the Rumen of Cows

**Figure 1:**
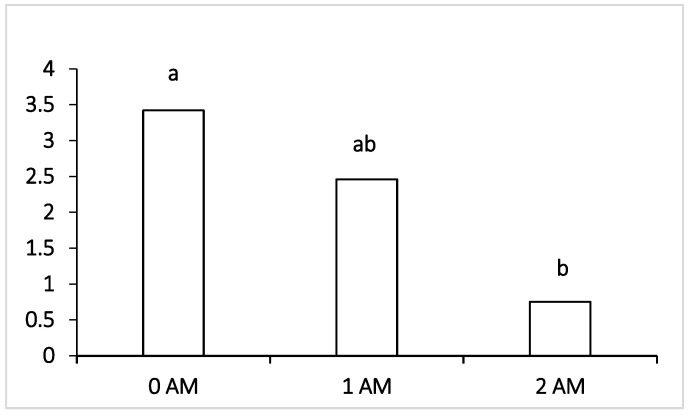
Effect of Agro-Matic supplementation on the rumen pathogenic microflora (*Fusobacterium* sp.).

**Figure 2:**
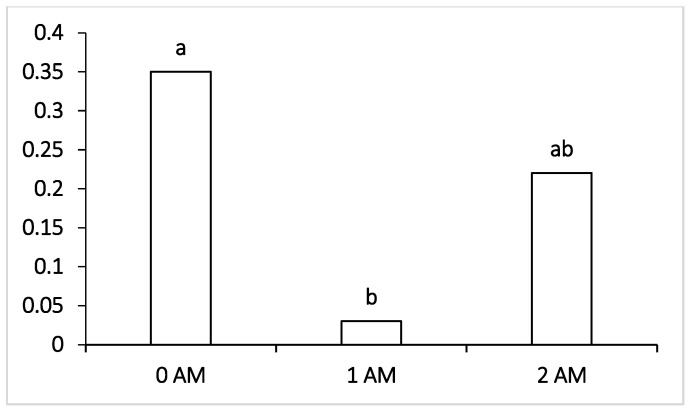
Effect of Agro-Matic supplementation on the rumen pathogenic microflora (*Peptococcus* sp.).

The analysis of the microflora content of the rumen of Ayrshire dairy cows in the study receiving protein concentrate Agro-Matic during the early lactation period (120 DIM) is presented in Table 6. There were no significant differences between the control and the Agro-Matic 1 AM and 2 AM supplemented groups in most of the rumen microbiota except in that of pathogenic microorganisms, such as Peptococcus (Figure 1) and fusobacteria (Figure 2); a significant decrease was noted between the control and the Agro-Matic 1 AM and 2 AM supplemented groups.

The amount of Bacteroidetes in cows of the 1 AM and 2 AM groups that received protein concentrate Agro-Matic in their rations was reduced by 1.5% and 2.9%, respectively, when compared to the 0 AM group (Table 6). With reference to the content of Selenomonadales that breaks down urea and volatile fatty acids, an increase of 1.7% and 4.1% was noted in the 1 AM and 2 AM groups, respectively, when compared to the control animals.

The experimental groups of animals showed no deviations from the normal standards for cellulolytic, amylolytic, transit and pathogenic bacteria. On the other hand, both the 1 AM and 2 AM groups revealed a decrease in the number of cellulolytic microorganisms fermenting the carbohydrates of the feed (starch and cellulose); in contrast, microbiota with antimicrobial activity that stimulate the animal immunity, such as *Bifidobacterium* sp. and *Bacillus* sp., were increased.

The concentration of cellulolytic bacteria in the experimental groups was higher than normal; nevertheless, their total number in the 2 AM group was less than in the 1 AM group of cows and the control also, which amounted to 32.4%, 41.1% and 44.44%, respectively. The inclusion of 1.0 kg of protein concentrate Agro-Matic in the ration of the 1 AM group increased the content of cellulolytic bacteria of the family Clostridiaceae and Thermoanaerobacteriaceae in the rumen content, and their number was within normal limits. The level of Clostridiaceae, Thermoanaerobacteriaceae and Ruminococcaceae in the 1 AM group was 2.5%, 0.3% and 1.8% higher than in the control group, respectively (Table 6).

Concerning amylolytic microflora, it was noted that the content of Succinivibrio bacteria in the 1 AM group was 0.7% higher than in the control one. On the other hand, the content of Bacteroidetes was 1.5% and 2.9% lower in the 1 AM and 2 AM groups in relation to the control, respectively.

With reference to the microflora stimulating the development of immunity, the 1 AM group recorded a higher content of Selenomonadales, *Bifidobacterium* and *Bacillus* when compared to the 0 AM group.

The number of pathogenic microorganisms of mastitis and other diseases (*Lactobacillus* sp., actinomycetes and enterobacteria) was lower in the animals of both the 1 AM and 2 AM groups receiving protein concentrate as part of their rations (Table 6) compared with the control cows.

Regarding pathogenic microorganisms such as *Fusobacterium* (Figure 1) and *Peptococcus* (Figure 2), a significant decline was observed in cows when the protein concentrate Agro-Matic was supplemented when compared with the 0 AM group.

The content of the microflora of the rumen of experimental cows at the end of the lactation period is presented in Table 7. The results of the study showed that the composition of the microbial community of the rumen of the cows under study was represented by a rich variety of microorganisms; however, the content of all microorganisms was within normal values.

The total number of cellulolytic bacteria at the end of lactation with the use of protein concentrate in animals of the 1 AM experimental group increased by 6.4% compared with the 0 AM group, and, with the introduction of 1.5 kg Agro-Matic, it increased by 4%. The content of bacteria more responsible for the breakdown of the complex carbohydrates of cellulose and hemicellulose (Lachnospiraceae, Ruminococaceae and Eubacteriaceae) decreased in the 1 AM group when compared with the control cows.

On the other hand, the level of Clostridiaceae responsible for the digestibility of feed protein and Thermoanaerobacteria increased by 1.38% and 7.17% when supplementing with protein concentrate Agro-Matic in the 1 AM group and 2 AM group, respectively, when compared with the control animals.

**Figure 3:**
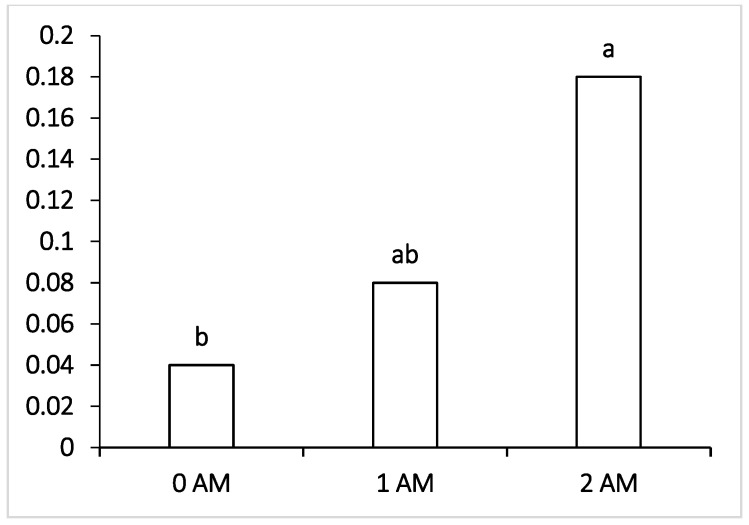
Effect of Agro-Matic supplementation on the rumen undesirable microflora (*Lactobacillus* sp.).

**Figure 4:**
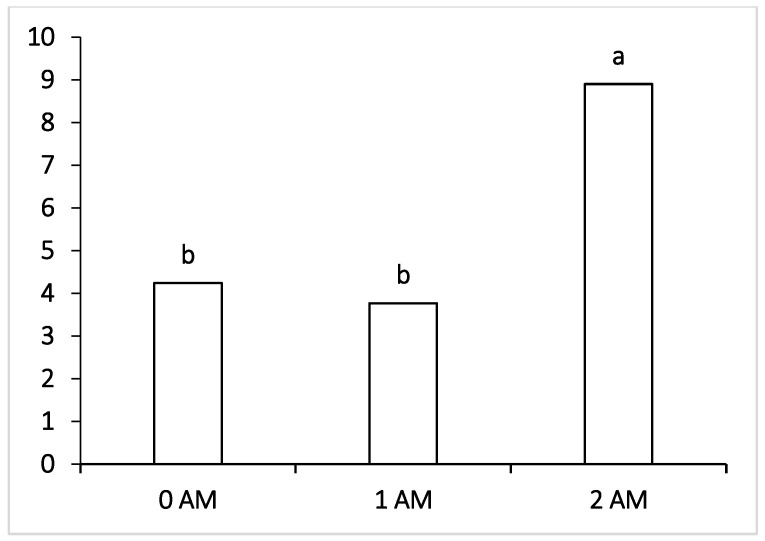
Effect of Agro-Matic supplementation on the rumen undesirable microflora (Actinobacteria).

Concerning undesirable bacteria, both the 1 AM and 2 AM groups recorded the highest value for *Lactobacillus* (Figure 3) and Actinobacteria (Figure 4), representing 0.18% and 8.90% vs. 0.04% and 4.24% in the 0 AM group, and the differences were significant (*p* < 0.05). On the other hand, there were no significant differences between the control group and the Agro-Matic 1 AM and 2 AM supplemented groups with regard to the other rumen microbiota.

The level of *Succinivibrio* bacteria in animals of the control group of cows was lower than in the rumen of animals of the 1 AM group. Concerning the level of Bacteroidetes in the 1 AM group and the 2 AM group: they increased and represented 9.32% and 8.72% vs. 6.78% in the 0 AM group. On the other hand, the number of Succinivibrio bacteria in the rumen of cows in the 0 AM and 1 AM groups was almost the same.

The level of Selenomonadales in the control group represented the highest value when compared with both the 1 AM and 2 AM groups, representing 9.58% vs. 7.88% and 4.74%, respectively. On the other hand, animals of both the 1 AM and 2 AM groups recorded the highest values for *Bacillus* and *Bifidobacterium* and represented 7.90% and 0.17% vs. 6.96% and 0.14% when compared with the 0 AM group, respectively.

Referring to the content of “unidentified” bacteria in the collected samples: it was also insignificant. The functional role of this group of microorganisms has not been determined; therefore, it was not subject to decoding. Meanwhile, a high level of “unidentified” bacteria was observed in animals from the 0 AM group, amounting to 18%.

There was an increase in the proportion of opportunistic and pathogenic microflora in the rumen community of the 0 AM group. The level of *Fusobacterium* was within normal values in the three groups of lactating cows. However, when the protein concentrate was supplemented in the feed of the 2 AM group, the level of *Fusobacterium* increased compared to the 0 AM group, and its highest value was recorded, amounting to 1.00% versus 0.24%.

Moreover, the number of *Staphylococcus* was within normal values; however, the lowest level (0.03%) was recorded for cows in the 1 AM group, while it increased slightly (0.16%) in the 2 AM group. The present results show that in both the 1 AM and 2 AM groups, the level of *Peptococcus* decreased, representing 0.11% and 0.03%, respectively, vs. 0.65% in the control animals. With reference to the level of Campylobacteriaceae: the cows fed with protein concentrate Agro-Matic did not show significant differences from the control group, and both 1 AM and 2 AM groups had levels amounting to 0.61% and 0.62% vs. 0.82% in the 0 AM group.

### 3.2. Reproductive Parameters of Lactating Cows

The effect of supplementing with protein concentrate Agro-Matic on some indicators of the reproductive ability of Ayrshire dairy cows is shown in Table 8.

The calving interval period in the control group when using traditional protein feed in the basic ration in animals of the 0 AM group was the longest and represented 407.05 vs. 383.00 and 377.79 for the 1 AM and 2 AM groups, respectively. In both the 1 AM and 2 AM groups, the insemination index (S/C) decreased when compared to the 0 AM group and represented 2.0 and 2.6, respectively, vs. 3.0.

With reference to the days open period, it was also noted that a shorter days open period was recorded in both the 1 AM and 2 AM groups when compared to the 0 AM group, representing 107.33 and 98.50, respectively, vs. 123.12.

### 3.3. Economic Efficiency of Milk Production

The results relating to the economic efficiency of the inclusion of protein concentrate in the diet are shown in Table 9.

The economic efficiency analysis was carried out at the end of the lactation period. At the same time, the costs of feed and feed additives were taken into account in the quotes of current prices for the duration of the experiment (2020–2021).

The inclusion of protein concentrate Agro-Matic increased the cost of daily ration by USD 0.23 and USD 0.20 in the 1 AM and 2 AM groups, respectively, when compared with the control group due to the cost of the introduced protein concentrate Agro-Matic. Moreover, the feed costs for the period of experiment recorded a difference *p* < 0.05 between the different experimental groups and represented USD 761.37 and USD 757.98 vs. USD 733.73 for the 1 AM, 2 AM and 0 AM groups, respectively. The high milk productivity of cows is determined by full-concentrated feeding, genetic potential, live weight, milking technology and the animal welfare system [47]. Thus, the cash obtained from the sale of milk in both the 1 AM and 2 AM groups increased by USD 89.65 and USD 159.37, respectively, when compared to the 0 AM group; on the other hand, the total milk production costs were higher in the supplemented groups 1 AM and 2 AM when compared to the control group, and the differences were significant. 

The profitability (%) was the lowest in the 0 AM group; however, with the introduction of protein concentrate Agro-Matic, it was increased by 2.76% and 6.28% in the 1 AM and 2 AM groups, respectively.

## 4. Discussion

### 4.1. Bacterial Community of the Rumen of Cows

Cellulolytic fermentation supplies the body of cows with substrates and ensures the digestibility of nutrients from indigestible to digested materials and transforms them into monomers with the help of microbial enzymes [48]. In general, the experimental groups of animals showed no deviations from the norm for cellulolytic, amylolytic, transit and pathogenic bacteria, which indicated balanced feeding. The introduction of Agro-Matic protein concentrate into rations caused a decrease in the number of cellulolytic microorganisms fermenting the structural carbohydrates of the plant feed [47,48,49,50] as microbiota with antimicrobial activity, it was noted a higher content of such as *Bifidobacterium* sp. and *Bacillus* sp. The observed depression of fiber digestion when adding protein concentrate Agro-Matic to the ration was a consequence of the negative associative effect of feed concentrate on ruminant activity and digestion. The level of cellulolytic bacteria in the experimental groups was higher than normal; however, the total sum in the 2 AM group was lower than that in the 1 AM group of lactating cows and the control group.

The effectiveness of the use of non-protein nitrogen by the rumen microflora for their own needs and protein synthesis of the body depends primarily on the ratio of sugar and protein in the ration [49]. It is known that if the dry matter of the animal diet contains 10–12% protein, then non-protein nitrogen is used well; if the protein is more than 14–16%, then the degree of assimilation of non-protein nitrogen decreases sharply. At the same time, for the development of microorganisms and the formation of protein, a sufficient number of easily digestible carbohydrates must be present in the feed [50,51].

At the beginning of the lactation period, calcium is poorly mobilized from the skeleton. As a result of intensive milk synthesis, the mobile supply of calcium is rapidly removed from the blood, disrupting regulatory functions. During this period, it is important that a sufficient level of magnesium is supplied to the ration. This element participates in the process of calcium mobilization from the cow’s backbone after calving. Magnesium deficiency makes it difficult to form a parathyroid hormone that mobilizes calcium. When analyzing the diet of cows by mineral nutrition, attention is paid to the ratio of calcium to phosphorus as part of the mineral metabolism of the body. To a greater extent, the ratio under consideration should be normalized in the diets of highly productive cows [52].

It is known that calcium ions are necessary for three predominant types of rumen cellulolytic bacteria, such as *Fibrobacter succinogenes*, *Ruminococcus flavefaciens* and *Ruminococcus albus*, which are present in the rumen fluid. Bacteria of the genus *Ruminococcus* probably synthesize cellulosomes that require the presence of calcium ions in the structure. The function of calcium in the degradation of cellulose by *F. succinogenes* is unknown, but it is also associated with the secretion or activation of cellulolytic enzymes [53].

When studying the microbiota of the rumen of cows at the end of the lactation period, we found that the inclusion of Agro-Matic protein concentrate in the rations directly affected the number of cellulolytic microorganisms fermenting structural carbohydrates of plant feed. Concerning the rumen microflora at the end of lactation, the sum of cellulolytic bacteria increased in all experimental groups. The highest level of cellulolytic was observed in animals of the 1 AM group. On the other hand, the decrease in the level of cellulolytic bacteria Lachnospiraceae and Ruminococcaceae in cows of the 1 AM group was probably due to a drop in the pH of the rumen content, as the concentration of these microorganisms largely depends on the acidity of the rumen, which is highly correlated with amylolytic microorganisms, such as Bacteroides and Succinivibrio, which are formed during the fermentation of concentrated feeds with a high level of starch.

It is known that Bacteroidetes are necessary for the ecosystem of the rumen of cows since they use starch of concentrated feed as a substrate for their vital activity and the formation of byproducts: lactic, acetic, propionic, succinic and butyric acids [52,53,54]. Additionally, in a study by Osborne and Dehority (1989) [54], it was suggested that some representatives of the Bacteroidetes phylum, such as Prevotella ruminicola, may synergistically participate in the degradation of plant cell walls in order to fully use feed cellulose, hemicellulose and pectin for conversion into volatile fatty acids. On the other hand, Bacteroidetes are responsible for the breakdown of protein [46] and starch in concentrated feed. In our study, in the experimental groups at the end of lactation period, a higher content of Bacteroidetes was observed. Therefore, the reduction in the number of this group in the control group was due to an increase in the level of available protein necessary for the growth of the number of bacteria. In this regard, it is possible to prove the increase in the intensity of protein metabolism in the rumen of the experimental lactating cows.

It was interesting to note that, at the beginning of lactation (120 DIM), both experimental groups (1 AM and 2 AM) revealed a lower content of Bacteroidetes compared to the control, amounting to 1.5% and 2.9%, respectively. The increase in the proportion of these bacteria noted at the end of lactation in animals of the experimental groups compared with the control group was probably due to the reorganization of the microbiome that occurred during lactation under the influence of the protein concentrate Agro-Matic. Interestingly, changes in the representation of bacteria of the order Selemonadales in the rumen of cows during the first period (120 DIM) and at the end of lactation had reverse trends of change compared with the content of Bacteroidetes. During the fermentation of starch and easily digestible carbohydrates, a high number of fermentation byproducts are formed—organic acids, among which is a high proportion of lactic acid, which is a substrate for bacteria of the Selenomonadales group. It has been reported that acid-utilizing bacteria of the genera Megasphaera, Selenomonas and Dialister are physiologically important groups for cattle, since they do not allow lactate to accumulate in the rumen, the increase in the proportion of which can lead to the decrease in pH and initiate the development of lactate acidosis [47,48]. At the beginning of the lactation period, the representation of these microorganisms in the rumen of cows of the experimental groups was higher compared to the control by 1.7% and 4.1%. At the end of lactation, the content of Selenomonadales was highest in cows of the control group, which is probably due to the presence of a large amount of substrate for their growth.

Obviously, the presence of lactate-producing Lactobacillus species was also associated with the number of Selenomonadales. It should be noted that Selenomonadales, like cellulolytic taxa, are sensitive to the decrease in pH, which may explain their slight decrease in the rumen with the increase in the proportion of Bacteroidetes at the end of the lactation period. There was a direct pattern of changes in the proportion of VFA-fermenting Selenomonadales compared with other microorganisms, particularly compared with the content of representatives of Clostridiaceae. It is known that many representatives of this family have proteolytic properties and participate in the fermentation of oligosaccharides, starch and sugar to form formate, acetate, lactate, propionate, butyrate and other volatile fatty acids, which provide most of the energy for the host [49].

Generally, throughout the course of our research, there was a modulation of the microbial community associated with the increase in the representation of the proportion of bacteria with protein, cellulose and amylolytic properties in the rumen of cows by the end of lactation when feeding with a feed additive. This potentially indicates the increase in the activity of the microbiome in relation to protein, polysaccharides and easily digestible feed components, which influenced the level of dairy productivity of cows in the study which were supplemented with the protein concentrate Agro-Matic. The VFA formed during the fermentation of carbohydrates, in particular, propionic, are used in the body for milk synthesis. The increase in the content of propionate can also stimulate insulin secretion, blood flow to the udder and milk protein synthesis [49,50]. Acetate and butyrate in the rumen are converted by the liver into cholesterol in order to further participate in the synthesis of milk fat [50,51].

In addition, short-chain fatty acids produced by bacteria have a number of other important properties. For example, they are involved in the epigenomic regulation of interactions between the microbiota and the host macroorganism [52,53,54,55,56,57,58]. It is also known that epigenetic modifications are able to regulate gene expression, affecting its intensity and duration, without changes in the main DNA sequence. Moreover, many VFA produced during the decomposition of feed carbohydrates have pronounced antimicrobial properties, contributing to a decrease in the representation of pathogenic microorganisms [59,60,61].

The symbiotic microflora of the rumen perform a detoxification function, regulate the digestibility of nutrients, prevent the invasion of parasites and pathogens and stimulate the development of immunity. The proof of this is that, according to the latest research data by Holman and Gzyl (2019) [62], the microflora and microfauna of the gastrointestinal tract of animals play an important role in the development and maturation of the immune system of cows [63] and can also provide protection of the digestive system from colonization by pathogenic microorganisms. In addition, the slow formation of the microbial community of the rumen occurs due to ecological and biological factors created by the microorganism, for example, the influence of the immune system of cows, which synthesizes immune peptides, glycolyzed animal cells and nutrients supplied to bacteria from the body of the cows.

We noted that the proportion of bacteria with antagonistic properties against pathogens in the rumen of animals increased under the action of protein concentrate Agro-Matic. The patterns of increased concentrations of bacteria that stimulate the development of immunity revealed by us in the initial period of lactation indicate a higher content of *Bifidobacterium* sp. and *Bacillus* sp. compared to the group 1 AM. However, at the end of lactation, animals of both groups 1 AM and 2 AM showed the highest values for Bacillus and Bifidobacterium. The saccharolytic properties of Bifidobacterium and its ability to produce acetate and lactate are also known [31,56,57,58,59].

When analyzing the representation of various opportunistic and transit microorganisms, it was found that most of them belonged to bacteria associated with the development of gastroenteritis—the families Enterobacteriaceae and Pseudomonadaceae. Interestingly, in the early period of lactation in the cows, the proportion of these microorganisms significantly decreased compared to the control cows that did not receive the protein concentrate Agro-Matic. In the late period, the proportion of these bacteria, as well as actinomycetes, increased, which may be due to their proteolytic activity [3]. It is known that the increase in opportunistic and pathogenic microflora in the rumen community is directly related to the decrease in the level of productivity and the state of animal health. The state of the rumen microflora directly affects the development of diseases of the liver, internal organs and limbs and mastitis and causes a decrease in reproduction rates, which was confirmed by some studies [64].

Regarding pathogenic microorganisms such as Fusobacteria, which can penetrate the blood and infect the body of cows with the development of liver abscesses and hoof lesions, their level was within the normal values in all the experimental groups of lactating cows receiving the ration with protein concentrate. The lowest content of pathogenic microorganisms, pathogens of mastitis and purulent-necrotic processes (*Staphylococcus, Peptococcus* and *Fusobacterium*) during the early lactation period (DIM = 120) was found in the ruminal fluid of animals of both the 1 AM and 2 AM groups that were supplemented with Agro-Matic protein concentrate as part of their diet. The number of pathogenic microorganisms—such as *Peptococcus*—which cause purulent-necrotic diseases was significantly lower in animals of the 1 AM group, and Fusobacteria causing necrobacteriosis was also significantly lower in the ruminal fluid of the 2 AM group of cows. It may be interpreted that fusobacteria are capable of lactate consumption, and the decrease in their number may be correlated with the decrease in the proportion of lactate-producing Bacteroidetes, Lactobacillus and the increase in the content of other lactate-utilizing bacteria—Selenomonadales. However, at the end of lactation period, the level of Fusobacteria increased and was the highest among the compared groups in the 2 AM group versus the 0 AM group; moreover, there was an increase in pathogenic *Clostridium*, which may be attributed to the fact that the advantage of protein feeds of animal origin is more associated with the participation of these groups of microorganisms. It is known that fusobacteria are characterized by high proteolytic activity [58], which could be the reason for their increase when the protein supplement was added to the ration.

The representation of pathogenic bacteria such as Staphylococcus, Peptococcus and Campylobacteriaceae in the rumen of cows of both experimental groups at the end of the lactation period was lower compared to the control group. This indicates that the introduction of a protein feed additive had a positive effect on the composition of the rumen microbiota, contributing to a decrease in the representation of pathogenic bacteria. Similar results were obtained in the studies of Holman and Gzyl (2019) [53], which reflects the influence of the microflora of the ruminant rumen on the development and maturation of the immune system of cows, including the protection of the digestive tract from colonization by pathogenic microorganisms.

Thus, the decrease in the abundance of representatives of the genera Campylobacter and Fusobacterium in animals in the experimental group was consistent with a decrease in the number of somatic cells in milk previously reported, since it has been proven [65,66,67,68,69] that these microorganisms are associated with the occurrence of mastitis in cattle. The observed decrease in the number of pathogens such as Campylobacteriaceae, Staphylococcus and Peptococcus indicates the potential impact of Agro-Matic on reducing the presence of these genera, which can reduce the incidence and their spread into the environment.

Generally, molecular genetic studies conducted on lactating cows at the early lactation stage (120 DIM) and at the end of the lactation period show that the state of the rumen microflora affects not only the efficiency of feed digestion but also the immunity, health status, productivity level and the period of productive longevity of animals. In addition, the results obtained were consistent with the studies and works of Jami et al. (2014) [3], who noted that the species composition of the rumen depends on the habitat of cows, the season of the year, the quality of feed, breed and individual characteristics of the development of the animal organism. Thus, our study showed that the composition of the bacterial community of the rumen of the studied cows was represented by a rich variety of microorganisms, the content of which was within normal values. At the same time, there were obvious shifts in the microbiome of the rumen of the cows under the influence of the protein concentrate Agro-Matic associated mainly with energy and protein metabolism.

### 4.2. Reproductive Ability of Lactating Cows

The efficiency of milk production depends not only on level of milk productivity but also on the health of animals and their reproductive ability. Unbalanced feeding and high milk productivity negatively affect the reproductive function of cows, and the period of infertility contributes to the retirement of highly productive animals and a decrease in the efficiency of milk production [70,71,72].

One of the important indicators characterizing the reproductive function of cows is the days open period. In the lactating cows that received protein concentrate as part of their rations, the calving intervals, days open period and the service/conception decreased in both the 1 AM and 2 AM groups compared with the 0 AM group. Consequently, supplementation of Agro-Matic protein concentrate where CP% = 18.00 and 18.20 in the 1 AM and 2 AM groups had a positive impact on reproduction indicators. Our results are opposite to those obtained by Clark et al. (1985) [70], who found that the conception rate to first service was reduced from 61% to 33% when the dietary protein was increased from 10% to 16%. Additionally, Jordan and Swanson (1979) [30] found that the calving-to-conception interval (days open) elongated when dietary crude protein was increased from 12.7% to 16.3%.

In the study of Schwab (2017) [72], he reported that the ammonia formed from non-protein nitrogen and degradable protein is used for synthesis of bacterial protein: the optimal amount of protein to satisfy the nitrogen needs of the rumen microorganisms in order to achieve maximum fiber digestion and microbial protein synthesis.

Consequently, a properly balanced intake of amino acids from microbial protein and the use of protein supplement made it possible to achieve protection from degradation in the rumen.

### 4.3. Economic Efficiency of Milk Production

The high level of milk productivity of lactating cows has a significant effect on the profitability of milk production. To a greater extent, the positive effect of high milk productivity is restrained due to the occurrence of additional costs caused by feed production, waste disposal, preventive veterinary measures, zootechnical measures and an increase in labor. The high milk productivity of cows is determined by full-concentrated feeding, genetic potential, live weight, milking technology and the animal welfare system. The inclusion of Agro-Matic protein concentrate in the 1 AM and 2 AM groups contributed to the increase in the profitability of milk production of lactating cows when compared to the control group even though the daily cost of the diet in the experimental groups 1 AM and 2 AM increased when compared with control when protein concentrate was introduced into the ration. A high economic value was recorded for the 1 AM and 2 AM groups in terms of the profitability despite the increased feeding rations cost and total milk production costs. This may be attributed to the increase in gross milk production in the early lactation period (120 DIM) in the 1 AM and 2 AM groups when compared with the control group.

## 5. Conclusions

Utilization of different levels of Agro-Matic protein concentrate revealed no deviations from the normal standards of cellulolytic, amylolytic, transit and pathogenic bacteria of rumen microbiota, as well as no effect on reproductive ability, while it significantly improved the profitability of the milk production process of Ayrshire dairy cows during the early lactation period (120 DIM).

## Figures and Tables

**Table 1:** Composition and nutritional value of the basic ration fed to the dairy cows during the experimental period.

Ingredients	Experimental Groups		
	0 AM	1 AM	2 AM
Mixed grass hay (kg)	0.5	0.5	0.5
Corn silage (kg)	7	7	7
Mixed grass haylage from cereals (kg)	7	7	7
Barley grain haylage (kg)	12	12	12
Beet molasses (kg)	1.5	1.5	1.5
Beet pulp (dry) (kg)	1.5	1.5	1.5
Soybean cake (kg)	1.0	1.0	1.0
Sunflower cake (36% CP) (kg)	1.5	0.5	-
Agro-Matic protein concentrate (kg)	-	1.0	1.5
Concentrated feed compound (kg)	11	10.5	10.5
By pass oil (kg)	0.3	0.3	0.3
Monocalcium phosphate (g)	130	130	130
Sodium chloride (g)	120	120	120
**Nutritional value of the rations of cows during the experimental period**			
Metabolizable energy, MJ	279.0	275.5	276.6
Dry matter (g)	23 800	23 400	23 800
Crude protein (g)	4074.3	4213.9	4325.1
Crude protein (%)	17.10	18.00	18.20
Digestible protein	3000.2	3163.7	3274.9
Degradable protein	2880.5	2725.4	2674.6
Undegradable protein	1202.1	1500.0	1662.1
Crude fiber	4001.4	3790.85	3699.35
Crude fat	935.8	907.3	902.9
Neutral detergent fiber (NDF)	8698.3	8216.4	8281.6
Acid detergent fiber (ADF)	4531.2	4375.2	4313.2
Starch	5420.4	5240.6	5236.4
Sugar	1665.7	1638.9	1630.8
Calcium	164.2	175.7	183.0
Phosphorus	126.0	125.3	126.1
Magnesium	54.0	52.9	52.8
Potassium	265.2	252.6	247.7

The rations formulation of cows during dry and lactation periods constructed according to the All-Russia Institute of Animal Husbandry, 2016 [36].

**Table 2:** Nutritional value of protein concentrate Agro-Matic/1 kg.

Indicators	Contents (g)
Metabolic energy, MJ	13.0
Dry matter content	932.0
**Nutritional value per 1 kg of dry matter**	
Crude protein	582.5
Degradable protein	186.4
Undegradable protein	396.1
Digestible protein	502.4
Crude fiber	27.0
Acid detergent fiber (ADF)	23.3
Neutral detergent fiber (NDF)	130.5
Starch	10.0
Sugar	30.0
Crude fat	106.3

**Table 3:** The amino acid composition of the Agro-Matic protein concentrate.

Amino Acids	Amount (g/100 g)
Methionine	0.59
Cystine	0.42
Lysine	2.08
Threonine	1.38
Arginine	4.12
Isoleucine	1.26
Leucine	2.40
Valine	1.84
Histidine	0.67
Phenylalanine	1.46
Glycine	7.20
Serin	1.99
Proline	4.92
Alanine	4.08
Tryptophan	0.23
Aspartic acid	3.61
Glutamic acid	6.45

**Table 4:** The content of macro and micro elements in Agro-Matic protein concentrate.

Indicators	Amount	Indicators	Amount
Macro Elements (g/kg)		Micro Elements (mg/kg)	
Sodium	6.0	Iron	799
Calcium	34.0	Copper	4.8
Phosphorus	10.3	Zinc	33
Potassium	3.7	Cobalt	0.3
Magnesium	1.2	Manganese	121
Sulfur	11.6	Molybdenum	0.9
		Iodine	3.38
		Selenium	1.0

**Table 5:** Primers for amplification of rumen microorganisms.

N.	Gene	Target	Name of the Primer	Primer Sequence
1	16S	Bacteria	63f/1087r	5′AGGCCTAACACATGCAAGTC-3′ (Cy5) 5′-CTCGTTGCGGGACTTACCCC-3′

**Table 6:** Percentages of rumen microbiota of Ayrshire dairy cows during the early lactation period (DIM = 120).

Group of Microorganisms	Experimental Groups			
	0 AM	1 AM	2 AM	SEM
Cellulolytic bacteria				
Lachnospiraceae	11.43	10.95	7.63	1.46
Ruminococcaceae	6.22	7.97	4.84	1.11
Eubacteriaceae	11.15	4.66	5.57	2.75
Clostridiaceae	6.11	8.61	8.28	0.78
Thermoanaerobacteriaceae	1.00	1.25	0.49	0.18
Amylolytic bacteria				
Bacteroidetes	8.43	6.88	5.51	0.91
*Succinivibrio* sp.	0.10	0.83	0.12	0.21
The sum of cellulolytic and amylolytic bacteria	44.44	41.14	32.44	3.51
Antagonistic bacteria				
Selenomonadales	12.42	14.08	16.47	1.89
*Bacillus* sp.	8.26	13.89	10.65	1.17
*Bifidobacterium* sp.	0.41	0.46	0.41	0.04
Undesirable bacteria				
*Lactobacillus* sp.	0.84	0.26	0.16	0.22
Enterobacteriaceae	9.72	1.62	5.23	1.75
Actinobacteria	9.12	7.16	7.24	0.85
Transit bacteria				
Pseudomonadaceae	0.03	6.90	3.64	1.47
Unidentified bacteria				
Unidentified bacteria	8.93	9.29	21.25	4.88
Pathogenic bacteria				
*Staphylococcus* sp.	1.15	1.51	1.13	0.30
Campylobacteriaceae	0.42	0.22	0.00 (UD)	0.10
Clostridium botulinum	1.10	1.55	1.16	0.30

0 AM = no Agro-Matic. 1 AM = 1.0 kg Agro-Matic/head/day. 2 AM = 1.5 kg Agro-Matic/head/day. Values are expressed as means. SEM = standard errors of the mean (the total SE of the three groups); (UD) = undetected; Min = minimum; max. = maximum.

**Table 7:** Percentages of rumen microbiota of Ayrshire dairy cows at the end of lactation period (DIM = 305 ds).

Group of Microorganisms		Experimental Groups			
		0 AM	1 AM	2 AM	SEM
Normal (Bacteria) Microflora					
Cellulolytic bacteria					
Lachnospiraceae		23.43	22.60	24.22	3.10
Ruminococcaceae		8.78	5.78	9.00	1.72
Eubacteriaceae		7.36	6.46	7.58	0.83
Clostridiaceae		5.88	7.26	3.89	0.77
Thermoanaerobacteriaceae		4.47	11.64	7.20	1.63
Amylolytic bacteria					
Bacteroidetes		6.78	9.32	8.72	1.39
*Succinivibrio* sp.		0.10	0.14	0.00	0.05
The sum of cellulolytic and amylolytic bacteria		56.80	63.20	60.75	1.88
Antagonistic bacteria					
Selenomonadales		9.58	7.88	4.74	1.17
*Bacillus* sp.		6.96	6.70	7.91	0.81
*Bifidobacterium* sp.		0.14	0.66	0.17	0.15
Undesirable bacteria					
Enterobacteriaceae		1.34	4.24	3.79	0.67
Transit bacteria					
Pseudomonadaceae		0.90	0.68	0.50	0.23
Unidentified bacteria					
Unidentified bacteria		17.96	11.83	11.02	1.79
Pathogenic bacteria					
*Staphylococcus* sp.		0.31	0.03	0.16	0.09
*Fusobacterium* sp.		0.24	0.19	1.00	0.24
*Peptococcus* sp.		0.65	0.11	0.03	0.23
Campylobacteriaceae		0.85	0.61	0.62	0.21
*Clostridium botulinum*		0.00	0.05	0.02	0.02

0 AM = no Agro-Matic. 1 AM = 1 kg Agro-Matic/head/day. 2 AM = 1.5 kg Agro-Matic/head/day. Values are expressed as means. SEM = standard errors of the mean.

**Table 8:** Reproductive parameters of Ayrshire lactating cows fed Agro-Matic protein concentrate at different levels.

Indicators	Experimental Groups			
	0 AM	1 AM	2 AM	SEM
Calving interval (days)	407.05	383.00	377.79	8.94
Days open period (days)	123.12	107.33	98.50	8.25
Service/conception (S/C)	3.00	2.60	2.00	0.22

0 AM = no Agro-Matic. 1 AM = 1 kg Agro-Matic/head/day. 2 AM = 1.5 kg Agro-Matic /head/day. Values are expressed as means. SEM = standard errors of the mean (the total SE of the three groups).

**Table 9:** The economic value of inclusion of Agro-Matic protein concentrate when feeding Ayrshire dairy cows during the early lactation period (head/USD).

Indicators	Experimental Groups			
	0 AM	1 AM	2 AM	SEM
The cost of a daily ration	6.11	6.34	6.31	0.06
protein concentrate (1 kg)	–	32.00	48.00	–
Feed costs for the period of experiment	733.73 ^b^	761.37 ^a^	757.98 ^a^	4.80
Feed costs per 1 kg of milk	0.19	0.18	0.17	0.23
Total milk production costs	1337.71 ^b^	1380.23 ^a^	1396.73 ^a^	10.09
Consumption of concentrates per 1 kg of milk, g	7.63	7.05	6.78	0.21
Production costs of 1 kg of milk of energy feed units	0.013	0.012	0.011	0.001
Milk yield *, kg/day	32.6	34.5	35.8	0.91
Gross milk yield *, kg (120 DIM)	3912.00 ^b^	4140.00 ^ab^	4296.00 ^a^	73.37
The selling price of 1 kg of milk	0.41	0.41	0.41	–
Cash obtained from the sale of milk	1623.60	1713.25	1782.97	33.20
Profit from the sale of milk	285.90 ^c^	333.02 ^b^	386.24 ^a^	15.62
The profitability, %	21.4 ^b^	24.1 ^ab^	27.7 ^a^	1.01
of milk production, %	–	+2.76	+6.28	–

* Productivity of cows is represented by milk of natural fat content. 0 AM = no Agro-Matic. 1 AM = 1 kg Agro-Matic/head/day. 2 AM = 1.5 kg Agro-Matic/head/day. Values are expressed as means. SEM = standard errors of the mean (The total SE of the three groups). Means denoted within the same row with different superscripts are significant (*p* < 0.05).

## Data Availability

The data presented in this study are available upon request from the corresponding author.
